# Movement Demands of Elite Under-20s and Senior International Rugby Union Players

**DOI:** 10.1371/journal.pone.0164990

**Published:** 2016-11-08

**Authors:** Daniel J. Cunningham, David A. Shearer, Scott Drawer, Ben Pollard, Robin Eager, Neil Taylor, Christian J. Cook, Liam P. Kilduff

**Affiliations:** 1 Applied Sport Technology Exercise and Medicine Research Centre (A-STEM), College of Engineering, Swansea University, Swansea, Wales; 2 School of Psychology and Therapeutic Studies, University of South Wales, Rhondda Cynon Taff, Wales; 3 Welsh Institute of Performance Science, College of Engineering, Swansea University, Swansea, Wales; 4 The Rugby Football Union, Greater London, England; Leeds Beckett University, UNITED KINGDOM

## Abstract

This study compared the movement demands of elite international Under-20 age grade (U20s) and senior international rugby union players during competitive tournament match play. Forty elite professional players from an U20 and 27 elite professional senior players from international performance squads were monitored using 10Hz global positioning systems (GPS) during 15 (U20s) and 8 (senior) international tournament matches during the 2014 and 2015 seasons. Data on distances, velocities, accelerations, decelerations, high metabolic load (HML) distance and efforts, and number of sprints were derived. Data files from players who played over 60 min (n = 258) were separated firstly into Forwards and Backs, and more specifically into six positional groups; FR–Front Row (prop & hooker), SR–Second Row, BR–Back Row (Flankers & No.8), HB–Half Backs (scrum half & outside half), MF–Midfield (centres), B3 –Back Three (wings & full back) for match analysis. Linear mixed models revealed significant differences between U20 and senior teams in both the forwards and backs. In the forwards the seniors covered greater HML distance (736.4 ± 280.3 vs 701.3 ± 198.7m, p = 0.01) and severe decelerations (2.38 ± 2.2 vs 2.28 ± 1.65, p = 0.05) compared to the U20s, but performed less relative HSR (3.1 ± 1.6 vs 3.2 ± 1.5, p < 0.01), moderate (19.4 ± 10.5 vs 23.6 ± 10.5, p = 0.01) and high accelerations (2.2 ± 1.9 vs 4.3 ± 2.7, p < 0.01) and sprint•min^-1^ (0.11 ± 0.06 vs 0.11 ± 0.05, p < 0.01). Senior backs covered a greater relative distance (73.3 ± 8.1 vs 69.1 ± 7.6 m•min^-1^, p < 0.01), greater High Metabolic Load (HML) distance (1138.0 ± 233.5 vs 1060.4 ± 218.1m, p < 0.01), HML efforts (112.7 ± 22.2 vs 98.8 ± 21.7, p < 0.01) and heavy decelerations (9.9 ± 4.3 vs 9.5 ± 4.4, p = 0.04) than the U20s backs. However, the U20s backs performed more relative HSR (7.3 ± 2.1 vs 7.2 ± 2.1, p <0.01) and sprint•min^-1^ (0.26 ± 0.07 vs 0.25 ± 0.07, p < 0.01). Further investigation highlighted differences between the 6 positional groups of the teams. The positional groups that differed the most on the variables measured were the FR and MF groups, with the U20s FR having higher outputs on HSR, moderate & high accelerations, moderate, high & severe decelerations, HML distance, HML efforts, and sprints•min^-1^. For the MF group the senior players produced greater values for relative distance covered, HSR, moderate decelerations, HML distance and sprint•min^-1^. The BR position group was most similar with the only differences seen on heavy accelerations (U20s higher) and moderate decelerations (seniors higher). Findings demonstrate that U20s internationals appear to be an adequate ‘stepping stone’ for preparing players for movement characteristics found senior International rugby, however, the current study highlight for the first time that certain positional groups may require more time to be able to match the movement demands required at a higher playing level than others. Conditioning staff must also bear in mind that the U20s players whilst maintaining or improving match movement capabilities may require to gain substantial mass in some positions to match their senior counterparts.

## Introduction

Rugby union is a high intensity intermittent sport, where periods of intense static exertions, collisions and running at various intensities are interspersed with random periods of lower intensity work and rest [1, 2]. Recent work has characterised the movement demands of senior professional rugby union players [1, 3–7]. There is, however, a lack of literature on movement demands, physical characteristics and match analysis at the highest level of rugby union (international), with only a few studies published on work:rest ratios [8, 9], endocrine response [10, 11], time motion analysis [12] and a recent publication on movement demands [13]. A study by Quarrie et al., [12] reported rugby union players covered on average between 5.5 and 6.3 km per game during 27 international matches observed in their study. Backs generally covered greater distances compared to the forwards, conversely forwards sustained greater contact loads from scrums, rucks and mauls. These researchers utilised video and player tracking software to quantify distances and contact elements of the game. Unfortunately the current micro-sensor technology appears inadequate in quantifying the collision based elements of the game [14]. A cluster analysis revealed 5 distinct groups of players (e.g. props, second rows, back row, wings & fullback (back 3), centres & fly half) with the authors suggesting that hookers should be grouped with props or second rows and not back row players, which has previously been the case in older time-motion studies [15–17]. Although positional groups covered similar distances during matches, the distances they covered at various speed zones varied considerably and the amount of game time also varied significantly across positions due to tactical substitutions [12]. They also suggested that there was a difference in the amount of high speed running (>5m•s^-1^) performed at international level compared to lower levels of the game and therefore, players hoping to compete at international level need to be conditioned for the increased intensity of match play [12].

In addition, there is little in the literature about the elite development pathways (e.g. U20s internationals). With the exception of the work by Lombard et al., [18] on the 10 year physical evolution of South African U20s players, the research of Barr and colleagues [19] reporting speed characteristics of U20s players from a nation outside of the top 10 (i.e. tier 2), and movement characteristics in U20 players However, currently no literature exists on how these demands map with those of their senior counterparts. This information would be useful in order to prepare players for the movement demands of senior rugby, whilst minimising the risk of injuries by monitoring playing/training ensuring acute:chronic workloads are appropriate [20].

There has been a historical evolution of both senior and junior rugby players from a physical perspective [18, 21] with the rate of increase in body mass particularly large in the last 30–40 years [21]. For example, South African U20s players have increased in height (~2.8%), weight (~14%), strength (~51%), muscular endurance (~50%), and improved speed times over 10m (~7%) and 40m (~4%) but have not improved aerobic performance over a 13 year period [18]. This change is most likely due to the amount of training time available since the advent of professionalism and the training method advances made within the domain of strength and conditioning coupled with the desire for larger rugby players in order to gain the upper hand in the collision/contact area of the game. Despite seniors and juniors showing rapid developments in physical characteristics there still appears to be differences between these groups. For example, at the 2015 6 Nations tournament (the highest level of international competition in the northern hemisphere) the average weight of a senior English forward was 115.8 ± 8.12 kg compared to 110.3 ± 8.14 kg for an U20s forward. Similarly for the backs 93.8 ± 6.9 vs 89.9 ± 5.7 kg for seniors and U20s (unpublished data). Argus and co-workers [22] also found moderate to very large differences in mass, upper and lower body strength and upper body and lower body power between academy and senior professional southern hemisphere rugby union players. Interestingly Barr et al., [19] found no significant differences between senior and U20s internationals for initial and maximal sprint velocity, however, when initial and maximal sprint momentum was calculated there were significant differences between the groups. These findings are further corroborated by the work of Hansen and colleagues [23] who reported significant differences between elite senior and junior players for mass and measures of strength and power, but not for speed times over 5, 10 and 30m.

In rugby league, significant differences in distance travelled during match play between professional senior and elite junior players have been reported by McClellan & Lovell [24]. Specifically, the mean total distance travelled during professional games (8371 ± 897 m) was significantly greater than that travelled during elite junior (4646 ± 978 m) match-play. Research indicates a progression of physical characteristics between playing levels in rugby league players, Gabbett [25] outlined the progressive improvement in the physiological capacities (mass, speed, agility and aerobic endurance) of rugby league players as the playing level increases from U13 right the way through to professional level. A similar progression in physiological characteristics was reported in an elite English rugby union academy, when Darrall-Jones et al., [26] undertook a comprehensive testing battery with the U16, U18 and U21 academy squads. They reported a progressive increase between the groups on mass, strength, power and momentum, however no differences were found on aerobic endurance or speed times. Recent work by Gannon et al [27] showed improvements in strength and power measures over the course of a season in a professional club environment. The largest improvements were seen in the early to mid-season period with a drop off towards the end of the season but still achieving an overall gain from the start of the season. Neither of these studies made any comparison between age grade players and seniors.

A greater understanding of player movement patterns in senior and U20s international rugby union, may give an indication of the positional requirements of performance (as shown in senior professional rugby by Lindsay et al., [3]). Which may also aid in targeting qualities/physical outputs players need to work on. This will facilitate the planning and implementation of training programmes and development pathways that elicit the required physiological adaptations specific to individual player needs, whilst ensuring increases in training/playing load are applied appropriately to minimise the risk of injury [20]. Conversely, it could help identify outstanding performers who could be fast tracked into the senior set up. Therefore, assessing how U20s compare to seniors and ascertaining where this development tool sits in the player pathway to senior international honours needs to be addressed. Therefore, the aim of this study was to compare U20s international competitions with senior international competitions based on movement demands recorded using GPS devices.

## Methods

Elite professional junior players from an U20s international performance squad (n = 43), and elite professional senior players from an international performance squad (n = 27) participated in the study. Prior to providing written informed consent, participants were given information outlining the rationale, potential applications and procedures associated with the study. Ethics approval was granted by the Swansea University Ethics Committee. All players were considered healthy and injury-free at the time of the study and were in full-time training. Players were grouped broadly as forwards and backs and more specifically in sub units within those groups. With front row (FR), second row (SR) and back row (BR) making up the forwards. Half backs (HB), midfield/centres (MF) and back three (B3) players making up the backs. The U20s players (Table 1) provided a total of 161 GPS files from 15 games from two 6 Nations tournaments (2014 and 2015) and the 2015 Junior World Cup. The senior players (Table 1) provided a total of 97 GPS files from 8 games from the 2014 and 2015 6 Nations tournaments. Previous studies have shown that substitute players display greater work-rates compared to players who start the match, suggesting that these players do not pace their involvement [28]. Therefore, to be included in the analysis players had to complete ≥60 mins match time [5, 29]. The seniors won all 8 games (5 home, 3 away), the U20 won 8/10 (5 home, 5 away) in the 6 Nations and 4/5 at the Junior World Cup (neutral venues). The average points scored per game was 31.3 and 36, and points conceded 15.4 and 11.4 for senior and U20 respectively. Each player provided at least 1 GPS file with the largest number of files provided by any one player being 11 and 8 in the U20s and seniors, respectively. A total of 79 GPS units were used during the study, units were returned to the manufacturer at the end of each competition for maintenance/repair.

**Table 1:** Anthropometric Characteristics of Position Groups.

Positional Group	Team	Age (years) M ± SD	Height (cm) M ± SD	Mass (kg) M ± SD
FR	Senior	26.1 ± 2.3	185.7 ± 4.2	119.1 ± 5.0
	U20	19.5 ± 0.7	184.4 ± 3.0	111.8 ± 5.6
SR	Senior	26.4 ± 3.3	199.2 ± 1.6	116.8 ± 4.8
	U20	19.7 ± 0.5	199.7 ± 2.3	115.2 ± 4.1
BR	Senior	26.0 ± 3.3	190.0 ± 2.6	117.7 ± 10.4
	U20	19.9 ± 0.3	187.7 ± 2.7	101.6 ± 3.9
HB	Senior	24.2 ± 2.5	179.5 ± 6.0	88.7 ± 4.6
	U20	19.6 ± 0.4	176.0 ± 2.1	84.2 ± 4.1
MF	Senior	25.7 ± 1.3	190.2 ± 4.1	102.3 ± 6.9
	U20	19.5 ± 0.6	183.0 ± 4.9	96.1 ± 6.6
B3	Senior	24.6 ± 3.4	182.6 ± 4.1	91.7 ± 2.1
	U20	19.6 ± 0.5	183.7 ± 4.3	89.6 ± 4.9

FR = Front Row (Prop & Hooker), SR = Second Row, BR = Back Row, HB = Half Backs, MF = Midfield/Centres, B3 = Back Three (Wing & Full Back).

### Procedures

All Matches took place between January 2014 and June 2015, each player wore a GPS unit (Viper Pod, STATSport, Belfast, UK) in a bespoke pocket incorporated into their playing jersey on the upper thoracic spine between the scapulae to reduce movement artefacts [30]. The GPS units captured data at a sampling frequency of 10Hz utilising the 4 best available satellites. Recent advancements in GPS technology have made 10 Hz units commercially available, which are more accurate for quantifying movement patterns in team sports [31, 32]. For example, Varley et al., [32] reported that a 10 Hz GPS unit was two to three times more accurate for instantaneous velocity during tasks completed at a range of velocities compared to a criterion measure, 6 times more reliable for measuring maximum instantaneous velocity and had a coefficient of variation less than or similar to the calculated smallest worthwhile change [33] during all phases of acceleration/deceleration. More specifically this brand of GPS devices has been used in team sports to assess movement demands during training and competitive matches [13, 34–39]. In our study, all participants were already familiarized with the devices as part of their day-to-day training and playing practices. Units were activated according to the manufacturer’s guidelines immediately prior to the pre-match warm-up (~30–60 minutes before kick-off), and to avoid inter-unit variation players wore the same GPS device for each match. Post match, timings from the game were (e.g. kick off, half time, sin binning etc) were entered into the software the raw data files were then processed and data for distance covered, and acceleration/deceleration events in pre-set zones were derived automatically by the software (Viper PSA software, STATSports, Belfast, UK).

### Locomotor Variables

The distance relative to playing time (m•min^-1^), high speed running (HSR) relative to playing time >18.1km•h^-1^ (the threshold used in numerous rugby GPS studies in both codes; e.g. Austin & Kelly [40] & Jones et al., [4]), number of sprints relative to playing time (sprints•min^-1^), moderate, high and severe intensity accelerations and decelerations (±2-3m•s^-2^, ±3-4m•s^-2^, ±>4m•s^-2^), high metabolic load distance (HML; defined as distance covered accelerating and decelerating over 2 m•s^-2^ and/or distance covered >5 m•s^-1^), and high metabolic load efforts (the number of separate movements/efforts undertaken in producing HML distance). Total time was calculated for ‘playing time’ only, that is, the time the player was on the playing field only, with time off the field (e.g., half time, periods on the bench/sin bin) removed from the data analysis. Time off during match play, such as injury time or video referee, was included in the study, because this was part of the game duration; hence ‘playing time’ may exceed the standard 80 minutes of match play.

### Data Analysis

Linear mixed models were used to examine each dependent variable for the interaction between teams in respect to positional types (forwards and backs) and groups (e.g., front rows, second rows etc.). To allow for the nested design of the data, random intercepts were modelled for participants (individual GPS measures), teams (i.e., U20 vs Senior) and competition (U20 6 Nations 2014, U20 6 Nations 2016, U20 Junior World Cup 2015, Senior 6 Nations 2014, Senior 6 Nations 2015). Attempts were made to also model for random slopes for the same variables but this resulted in over-specified models. Where significant interactions were identified, differences were interpreted using a combination of estimates of fixed effects, examination of means and 95% confidence intervals (Tables C and D in S1 File).

## Results

Examination of means and standard deviations indicated visible difference between teams as a function of position type (Table 2). Linear mixed models indicated a significant interaction between Team (U20 v Senior) and Positional Type (Forwards and Backs) for M•min^-1^ (p < .001), HSR m•min^-1^ (p < .001), Accelerations 2-3m•s^-2^ (p < .01), Accelerations 3-4m•s^-2^ (p < .001), Decelerations 3-4m•s^-2^ (p < .001), Decelerations >4m•s^-2^ (p < .01), HML Distance (m) (p < .001), Sprint•min^-1^ (p < .001), and HML Efforts (p < .001). Further examination of fixed effects for these significant interactions revealed that U20 forwards had significantly higher HSR m•min^-1^ (p < .001, CI: -3.33 to– 1.08), Accelerations 2-3m•s^-2^ (p < .01, CI: -9.99 to– 1.46), Accelerations 3-4m•s^-2^ (p < .001, CI: -4.45 to– 1.72), Decelerations 3-4m•s^-2^ (p < .05, CI: -3.87 to– 0.44), Sprint•min^-1^ (p < .001, CI: -.12 to– 0.04), and significantly lower Accelerations >4m•s^-2^ (p < .05, CI: 0.13 to 3.66) and HML Distance (p < .05, CI: -292.12 to– 41.40). For backs, fixed effect revealed U20 players had significantly lower values for M•min^-1^ (p < .001, CI: 4.52 to 11.34), HML Distance (p < .001, CI: 140.65 to 402.96) HML efforts (p < .001, CI: 10.74 to 34.20) and Decelerations 3-4m•s^-2^ (p < .05, CI: 0.13 to 3.66), while significantly higher values for HSR m•min^-1^ (p < .001, CI: 1.15 to 3.51) and Sprint•min^-1^ (p < .001, CI: 0.04 to 0.13). However, some other variables were close to significance also (Table C in S1 File).

**Table 2:** Movement Characteristics for Senior and U20s, Forwards and Backs Groups.

Position Group	Forwards		Backs	
	Seniors (n = 15)	U20s (n = 21)	Seniors (n = 12)	U20s (n = 22)
GPS Variable	M ± SD	M ± SD	M ± SD	M ± SD
M•min^-1^	66.8 ± 7.0	61.5 ± 8.0	73.3 ± 8.1*	69.1 ± 7.6
HSR m•min^-1^	3.1 ± 1.6	3.2 ± 1.5^	7.2 ± 2.1	7.3 ± 2.1*
HML Distance (m)	736.4 ± 280.3^	701.3 ± 198.7	1138.0 ± 233.5*	1060.4 ± 218.1
HML Efforts	84.8 ± 30.4	78.8 ± 21.5	112.7 ± 22.2*	98.8 ± 21.7
Accelerations 2-3m•s^-2^	19.42 ± 10.5*	23.6 ± 8.9^	26.4 ± 8.4	26.1 ± 10.1
Accelerations 3-4m•s^-2^	2.2 ± 1.9*	4.3 ± 2.7^	4.9 ± 3.0*	6.4 ± 4.5
Accelerations >4m•s^-2^	0.69 ± 0.95	0.47 ± 0.84	1.04 ± 1.22	0.89 ± 1.37
Decelerations 2-3m•s^-2^	24.56 ± 11.5	25.2 ± 9.3	28.4 ± 7.7*	25.3 ± 9.3
Decelerations 3-4m•s^-2^	6.4 ± 4.0	7.5 ± 3.5^	9.9 ± 4.3*	9.5 ± 4.4
Decelerations >4m•s^-2^	2.38 ± 2.2^	2.28 ± 1.65	4.39 ± 2.77	4.95 ± 3.0
Sprint•min^-1^	0.11 ± 0.06	0.11 ± 0.05^	0.25 ± 0.07	0.26 ± 0.07*

^ = Significantly higher than either Senior or U20 forwards counterpart.

* = Significantly higher than either Senior or U20 backs counterpart.

Examination of means and standard deviations indicated visible difference between teams as a function of positional groups (Table 3). Linear mixed models indicated a significant interaction between team (U20 v Senior) and positional groups (e.g., half-back, second rows etc.) for M•min^-1^ (p < .05), Accelerations 2-3m•s^-2^ (p < .05), Decelerations 2-3m•s^-2^ (p < .001), Decelerations 3-4m•s^-2^ (p> .05), HML Distance (p < .01), and HML efforts (p > .001). Estimates of fixed effects were used to indicate differences between specific playing group between teams where interactions occurred. U20 Front Rows scored significantly higher than seniors for HSR m•min^-1^ (p < .001, CI: -5.15 to -1.61), Accelerations 2-3m•s^-2^ (p < .001, CI: -21.08 to -8.09), Accelerations 3-4m•s^-2^ (p < .001, CI: -6.35 to -1.84), Decelerations 2-3m•s^-2^ (p < .001, CI: -17.78 to -6.79), Decelerations 3-4m•s^-2^ (p < .001, CI: -7.55 to -2.20), Decelerations >4m•s^-2^ (p < .01, CI: -4.04 to -0.59), HML Distance (p < .001, CI: -618.18 to -240.92), HML efforts (p < .001, CI: -51.90 to -20.07) and Sprint•min^-1^ (p < .001, CI: -0.20 to -0.07). U20 Second rows scored significantly higher for HSR m•min^-1^ (p < .05, CI: -3.92 to -0.13), Accelerations 3-4m•s^-2^ (p < .01, CI: -5.72 to -0.91), however seniors performed more Sprint•min^-1^ (p < .001, CI: -0.20 to -0.07). U20 Back rows had significantly less Decelerations 2-3m•s^-2^ (p < .01, CI: 1.74 to 11.46) but more Accelerations 3-4m•s^-2^ (p < .05, CI: -4.26 to -0.14). U20 Half back had significantly lower scores for for M•min^-1^ (p < .001 CI: 6.48 to 17.80), Decelerations 2-3m•s^-2^ (p < .05, CI: 0.20 to 11.86), HML Distance (p < .05, CI: 56.47 to 503.78) and HML efforts (p < .001, CI: 19.72 to 55.84). U20 Midfield players had significantly lower values for M•min^-1^ (p < .05, CI: 1.27 to 12.34), HSR m•min^-1^ (p < .001, CI: 1.05 to 5.05), Decelerations 2-3m•s^-2^ (p < .05, CI: 0.21 to 11.73), HML Distance (p < .01, CI: 122.37 to 543.28) and HML efforts (p < .01, CI: 7.07 to 41.74), Sprint•min^-1^ (p < .001, CI: 0.04 to 0.18), but higher values for Decelerations 3-4m•s^-2^ (p < .05, CI: 0.24 to 5.99). Finally, U20 Back Three players had significantly lower values for M•min^-1^ (p < .05, CI: 1.12 to 10.58), and HML Distance (p < .01, CI: 51.45 to 399.61), but higher values for HSR m•min^-1^ (p < .001, CI: 0.81 to 4.09), Decelerations >4m•s^-2^ (p < .05, CI: 0.20 to 3.39) and Sprint•min^-1^ (p < .001, CI: 0.04 to 0.15).

**Table 3:** Movement Characteristics Presented by Playing Groups.

Position Group	FR		SR		BR		HB		MF		B3	
	Team	M ± SD	Team	M ± SD	Team	M ± SD	Team	M ± SD	Team	M ± SD	Team	M ± SD
M•min^-1^	U20s	60.1 ± 7.2	U20s	60.8 ± 5.9	U20s	63.2 ± 9.7	U20s	67.5 ± 9.1*	U20s	70.5 ± 6.8*	U20s	68.7 ± 7.6*
	Seniors	61.1 ± 7.9	Seniors	67.6 ± 6.5	Seniors	69.9 ± 4.3	Seniors	77.4 ± 5.6	Seniors	71.9 ± 10.0	Seniors	70.8 ± 7.1
HSR m•min^-1^	U20s	2.5 ± 1.3*	U20s	3.0 ± 1.1*	U20s	4.0 ± 1.6	U20s	5.5 ± 2.4	U20s	7.2 ± 1.7*	U20s	8.1 ± 1.7*
	Seniors	1.8 ± 1.1	Seniors	2.9 ± 1.2	Seniors	4.0 ± 1.4	Seniors	6.3 ± 1.6	Seniors	8.0 ± 2.3	Seniors	7.4 ± 2.2
HML Distance (m)	U20s	584.9 ± 199.1*	U20s	673.3 ± 124.1	U20s	820.6 ± 182.5	U20s	954.8 ± 304.0*	U20s	1103.4 ± 168.6*	U20s	1069.7 ± 203.0
	Seniors	452.3 ± 172.9	Seniors	747.1 ± 227.9	Seniors	911.4 ± 210.2	Seniors	1144.5 ± 175.9	Seniors	1205.4 ± 265.6	Seniors	1076.1 ± 246.0
HML Efforts	U20s	66.0 ± 22.6*	U20s	77.2 ± 14.5	U20s	90.7 ± 19.0	U20s	99.9 ± 27.2*	U20s	105.0 ± 15.9	U20s	93.4 ± 22.4
	Seniors	54.6 ± 19.3	Seniors	85.8 ± 26.7	Seniors	103.4 ± 22.4	Seniors	126.3 ± 14.2	Seniors	113.9 ± 23.1	Seniors	99.7 ± 20.5
Accelerations 2-3m•s^-2^	U20s	17.8 ± 6.6*	U20s	22.9 ± 7.4	U20s	29.0 ± 8.5	U20s	23.5 ± 13.6	U20s	27.4 ± 10.3	U20s	26.1 ± 8.1
	Seniors	10.0 ± 6.0	Seniors	21.3 ± 9.4	Seniors	24.4 ± 9.5	Seniors	26.8 ± 7.9	Seniors	28.5 ± 9.4	Seniors	24.4 ± 7.9
Accelerations 3-4m•s^-2^	U20s	3.5 ± 2.4*	U20s	3.8 ± 2.1*	U20s	5.5 ± 3.1*	U20s	4.3 ± 5.4	U20s	5.9 ± 2.8	U20s	7.6 ± 4.9
	Seniors	1.1 ± 1.3	Seniors	1.8 ± 1.9	Seniors	3.1 ± 2.0	Seniors	4.8 ±2.9	Seniors	4.0 ± 3.0	Seniors	5.7 ± 3.0
Accelerations >4m•s^-2^	U20s	0.39 ± 0.75	U20s	0.25 ± 0.53*	U20s	0.71 ± 1.04	U20s	0.33 ± 0.49	U20s	0.45 ± 0.78	U20s	1.47 ± 1.73
	Seniors	0.50 ± 0.65	Seniors	0.83 ± 1.19	Seniors	0.73 ± 0.98	Seniors	1.19 ± 1.22	Seniors	1.07 ± 1.44	Seniors	0.89 ± 1.08
Decelerations 2-3m•s^-2^	U20s	21.1 ± 9.0*	U20s	24.8 ± 9.8	U20s	28.8 ± 7.9*	U20s	24.7 ± 9.9*	U20s	28.1 ± 8.9*	U20s	23.3 ± 9.1
	Seniors	13.0 ± 6.1	Seniors	24.5 ± 10.4	Seniors	32.0 ± 8.4	Seniors	31.1 ± 5.6	Seniors	31.1 ± 8.6	Seniors	23.9 ± 6.7
Decelerations 3-4m•s^-2^	U20s	6.2 ± 3.7*	U20s	8.0 ± 3.0	U20s	8.2 ± 3.4	U20s	6.5 ± 3.6	U20s	11.5 ± 4.1*	U20s	9.2 ± 4.3
	Seniors	3.5 ± 2.4	Seniors	5.7 ± 2.4	Seniors	8.6 ± 4.3	Seniors	9.4 ± 4.8	Seniors	11.3 ± 4.2	Seniors	9.2 ± 3.9
Decelerations >4m•s^-2^	U20s	2.2 ± 1.9*	U20s	1.6 ± 1.3	U20s	2.9 ± 1.5	U20s	3.0 ± 2.0	U20s	5.2 ± 3.5	U20s	5.5 ± 2.6*
	Seniors	1.0 ± 1.5	Seniors	2.7 ± 2.2	Seniors	3.1 ± 2.3	Seniors	3.7 ± 2.3	Seniors	4.3 ± 2.7	Seniors	5.1 ± 3.1
Sprint•min^-1^	U20s	0.09 ± 0.04*	U20s	0.10 ± 0.03*	U20s	0.14 ± 0.05	U20s	0.18 ± 0.06	U20s	0.27 ± 0.06*	U20s	0.29 ± 0.06*
	Seniors	0.06 ± 0.04	Seniors	0.11 ± 0.04	Seniors	0.14 ± 0.05	Seniors	0.21 ± 0.06	Seniors	0.28 ± 0.07	Seniors	0.27 ± 0.08

FR = Front Row (Prop & Hooker), SR = Second Row, BR = Back Row, HB = Half Backs, MF = Midfield/Centres, B3 = Back Three (Wing & Fullback).

* = significant difference to Senior counterpart

## Discussion

The aim of this study was to compare the locomotor demands of senior international and age grade international (U20) rugby union matches using GPS devices. The current study is the first to present an analysis of movement demands of senior international competition in comparison to the elite junior international competition. The results of the present study increase our understanding of the movement demands of competition experienced by players in existing international rugby union development pathways and determine whether the U20s competition reflects the movement demands of senior match-play. Therefore, the results of the current study may have implications for the design and implementation of physical conditioning programmes in order to prepare players for the movement demands of senior international rugby.

In general, the seniors covered greater relative distance for both forwards (66.8 ± 7.1 vs 61.5 ± 8.0m•min^-1^) and backs (73.3 ± 8.1 vs 69.1 ± 7.6m•min^-1^), however this was only statistically significant for the backs. The U20s forwards performed more HSR m•min^-1^ accelerations in zones 2–3 & 3-4m•s^-2^, decelerations 3-4m•s^-2^ and sprint•min^-1^ than the seniors, but less HML distance. In the Backs, the senior group covered more relative distance (m•min^-1^) performed more decelerations 3-4m•s^-2^, more HML distance & efforts, but the U20s performed more HSR m•min^-1^ and sprint•min^-1^. The U20s also had significantly longer match time, which could be due to different substitution strategies or a number of other factors (e.g. more injury stoppages, discipline issues, third match official use). The relative distance values presented in the current study are lower than a recent publication from a southern hemisphere club team [3] (Forwards: 77.3 ± 20.5, Backs: 84.7 ± 10.4m•min^-1^), and in between values produced by 2 different Pro 12 clubs [5, 7] (Forwards: 60.4 ± 7.8 & 71.6 ± 10.1, Backs: 67.8 ± 8.2 & 81.0 ± 10.2m•min^-1^). However, when comparing to data published from the Premiership (Forwards: 64.6 IQR 6.3, Backs: 71.1 IQR 11.7 m•min^-1^) from which the current players are drawn, it appears that U20s international competition is marginally below the movement demands of the Premiership, while senior international competition is higher, in terms of relative distance covered. This may indicate that U20s rugby is preparing players for movement demands in Premiership rugby, which in turn will help prepare for full international matches. However, given the likely variation in tactics/playing styles between the teams and the opposition faced, care must taken when making comparisons [41, 42].

Although generally there are differences between senior and U20s backs and forwards, the number of variables that were significantly different varied across each positional group. There were no significant differences in relative distance covered between U20s and seniors in any forward positional group (front row, second row, back row). There were significant differences in relative distance covered for the half backs (77.37 ± 5.62 vs 67.47 ± 9.10m•min^-1^), midfield (71.9 ± 10.0 vs 70.5 ± 6.8m•min^-1^) and back three (70.8 ± 7.1 vs 68.7 ± 7.6m•min^-1^) with seniors covering greater distances in all cases. The U20s covered greater relative HSR distance in the front & second rows and back three position groups, with the opposite being the case for the midfield group. No differences between seniors and U20s were seen for back row or half backs for HSR.

The front row and midfield groups had the most differences between seniors and U20s. Significant differences were found between front row groups on HSR (relative), accelerations (2–3 and 3-4m•s^-2^), decelerations (2–3, 3–4 and >4m•s^-2^), HML distance, HML efforts, and sprints•min^-1^ with the U20s having higher outputs on each variable. Conversely for the midfield group seniors had significantly greater values for relative distance covered, HSR (relative), decelerations (2–3•s^-2^), HML distance and sprints•min^-1^. The back row group was most similar with only accelerations (2–3•s^-2^), decelerations (2–3•s^-2^) being significantly different. Comparing acceleration and deceleration data with previous literature is somewhat problematic as Cunniffe et al. [6] reported no differences between backs and forwards groups, however, a very low sample size was utilised (1 back, 1 forward during 1 game) together with different acceleration zones. Jones et al., [5] reported distance covered while in various acceleration and deceleration zones for both backs and forwards combined when investigating temporal fatigue in their study, which makes comparison impossible, as the current study used number of acceleration and deceleration events. Owen and co-workers [43] utilised comparable zones and reported number of acceleration/deceleration events, their work supports the current finding that backs are involved in more frequent acceleration and deceleration events. However, the current study appears to have a slightly higher frequency for both backs and forwards, potentially as a result of the level of competition (Super vs International rugby).

The number of sprints•min^-1^ performed was significantly different between U20s and senior forwards (0.11 ± 0.05 vs 0.11 ± 0.06) and U20s and senior backs (0.26 ± 0.07 vs 0.25 ± 0.07). However, this is unlikely to be of practical significance given the low frequency. Backs performed more sprints than the forwards (~x2.5) in both groups. The greatest difference between positional groups was the U20s front row group who performed almost double the amount of their senior counterparts (8.73 ± 4.52 vs 4.71 ± 3.45). This could be due to U20s players being lighter (119.1 ± 5.0 vs 111.8 ± 5.6 kg) and potentially more mobile, or simply a reflection of their physical capabilities. The number of sprints reported in the current study is higher than those reported by Jones and co-workers [5] most likely again to the difference in playing standard (club vs international) or potentially style of play.

Overall there were a number of differences between the forwards and backs of the U20s and senior teams. However, when broken down further into positional groups, variations in differences between the two teams in certain positions emerged. The positional groups that appeared most different between the teams on the metrics measured were the front row and midfield groups, with the U20s front row performing more than their senior counterparts on HSR, moderate and heavy accelerations, all decelerations, HML distance HML efforts. As the senior players tend to be heavier and stronger, the static exertions (not measureable by GPS) from scrums have been shown to be greater in the senior international game [44]. This may result in transient fatigue, whereby there is a reduction in high-intensity activity performed immediately following an intense bout, with a subsequent recovery later in performance [45], and account for their lower movement scores. The opposite was true for the midfield group with the seniors producing higher scores for relative distance covered, HSR, moderate decelerations, HML distance and sprints•min^-1^. Perhaps indicating these players are used more frequently in a more direct, gain-line based game plan.

High speed running (HSR) has previously been shown to distinguish between playing levels in a number of sports (e.g. [12, 46, 47]), with the more elite levels covering greater distances in this speed zone. However, in the current study overall the U20s forwards and backs groups, performed more relative HSR than their senior counterparts. This wasn’t the case for each positional group however, both senior and U20 back row and half backs had no differences between them for HSR. Whilst the senior midfield group outperformed the U20s. One potential reason for these discrepancies is that the two groups used here (senior and U20) are not elite and non-elite as used in studies where HSR has been a distinguishing factor. Both groups could be viewed as ‘elite’, in support of this 8 players from the U20s cohort have already progressed to the senior squads. It is also worth noting U20s players generally weigh less than their senior counterparts so will need to be able to maintain the same movement work load (e.g. distance covered, HSR distance, accelerations, decelerations) whilst increasing in mass to prepare for senior internationals. To our knowledge this is the first study comparing movement demands of U20 and senior International rugby union matches. The data suggests that the movement demands in Under 20s internationals are adequate for preparing players for movement demands reported in International rugby. However, certain positional groups might require more work and/or time to match their senior counterparts than others. Conditioning staff must also bear in mind that the U20s players whilst maintaining or improving match movement capabilities may require to gain substantial mass in some positions to match their senior counterparts.

## Supporting Information

S1 File **Table A. Supplementary Movement Characteristics for Senior and U20s, Forwards and Backs Groups.** Data presented as Mean ± S.D **Table B. Supplementary Movement Characteristics Presented by Playing Groups.** FR = Front Row (Prop & Hooker), SR = Second Row, BR = Back Row, HB = Half Backs, MF = Midfield/Centres, B3 = Back Three (Wing & Fullback). Data presented as Mean ± S.D. **Table C. Estimates of fixed effects for all GPS variables displaying difference for position type between U20s and Seniors Table D. Estimates of fixed effects for all GPS variables displaying difference for position group between U20s and Seniors**
(DOCX)
