# Supplementary material for: Movement Demands of Elite Under-20s and Senior International Rugby Union Players
Source: PLoS One. 2016 Nov 8;11(11):e0164990. doi: 10.1371/journal.pone.0164990 (PMC5100986; doi:10.1371/journal.pone.0164990)
Supplement: S1 File — Table A. Supplementary Movement Characteristics for Senior and U20s, Forwards and Backs Groups. Data presented as Mean ± S.D Table B. Supplementary Movement Characteristics Presented by Playing Groups. FR = Front Row (Prop & Hooker), SR = Second Row, BR = Back Row, HB = Half Backs, MF = Midfield/Centres, B3 = Back Three (Wing & Fullback). Data presented as Mean ± S.D. Table C. Estimates of fixed effects for all GPS variables displaying difference for position type between U20s and Seniors Table D. Estimates of fixed effects for all GPS variables displaying difference for position group between U20s and Seniors (DOCX) [file pone.0164990.s001.docx]

**Table A. Supplementary Movement Characteristics for Senior and U20s, Forwards and Backs Groups**

| Position Group | Forwards | | Backs | |
| --- | --- | --- | --- | --- |
|  | Seniors (n=15) | U20s (n=21) | Seniors (n=12) | U20s (n=22) |
| GPS Variable | M ± SD | M ± SD | M ± SD | M ± SD |
| Total Distance (km) | 5.55 ± 0.96 | 5.37 ± 0.83 | 6.36 ± 0.81 | 6.23 ± 0.80 |
| HSR (m) | 263.1 ± 147.0 | 284.2 ± 134.9 | 629.7 ± 204.2 | 656.9 ± 182.7 |
| Duration (min) | 83.0 ± 9.9 | 87.6 ± 9.7 | 87.0 ± 7.6 | 90.4 ± 8.1 |
| Sprints | 10.67 ± 6.40 | 11.15 ± 5.06 | 25.10 ± 8.39 | 26.44 ± 7.47 |

Data presented as Mean ± S.D.

**Table B. Supplementary Movement Characteristics Presented by Playing Groups**

| Position Group | FR | | SR | | BR | | HB | | MF | | B3 | |
| --- | --- | --- | --- | --- | --- | --- | --- | --- | --- | --- | --- | --- |
|  | Team | M ± SD | Team | M ± SD | Team | M ± SD | Team | M ± SD | Team | M ± SD | Team | M ± SD |
| Total Distance (km) | U20s | 4.97 ± 0.75 | U20s | 5.41 ± 0.48 | U20s | 5.67 ± 0.98 | U20s | 5.84 ± 0.89 | U20s | 6.51 ± 0.71 | U20s | 6.18 ± 0.77 |
|  | Seniors | 4.63 ± 0.63 | Seniors | 5.78 ± 0.83 | Seniors | 6.02 ± 0.79 | Seniors | 6.45 ± 0.64 | Seniors | 6.24 ± 1.01 | Seniors | 6.38 ± 0.81 |
| HSR (m) | U20s | 211.6 ± 112.7 | U20s | 265.3 ± 94.2 | U20s | 359.7 ± 142.7 | U20s | 476.1 ± 204.1 | U20s | 661.7 ± 145.1 | U20s | 728.4 ± 150.2 |
|  | Seniors | 137.4 ± 80.4 | Seniors | 257.3 ± 124.5 | Seniors | 346.2 ± 135.8 | Seniors | 529.4 ± 150.8 | Seniors | 688.7 ± 203.8 | Seniors | 669.7 ± 221.8 |
| Duration (min) | U20s | 83.2 ± 10.9* | U20s | 89.3 ± 7.5 | U20s | 90.0 ± 9.1 | U20s | 87.0 ± 10.6 | U20s | 92.5 ± 6.4 | U20s | 90.1 ± 7.9 |
|  | Seniors | 75.9 ± 5.9 | Seniors | 85.6 ± 10.1 | Seniors | 86.1 ± 9.8 | Seniors | 83.7 ± 8.6 | Seniors | 87.0 ± 8.3 | Seniors | 90.0 ± 4.6 |
| Sprints | U20s | 8.7 ± 4.5 | U20s | 10.0 ± 3.3 | U20s | 14.1 ± 5.3 | U20s | 17.8 ± 6.5 | U20s | 27.9 ± 6.3 | U20s | 28.9 ± 6.1 |
|  | Seniors | 4.7 ± 3.5 | Seniors | 10.8 ± 5.3 | Seniors | 14.4 ± 5.6 | Seniors | 19.9 ± 6.5 | Seniors | 27.9 ± 7.7 | Seniors | 27.4 ± 8.7 |

FR = Front Row (Prop & Hooker), SR = Second Row, BR = Back Row, HB = Half Backs, MF = Midfield/Centres, B3 = Back Three (Wing & Fullback). Data presented as Mean ± S.D.

**Table C. Estimates of fixed effects for all GPS variables displaying difference for position type between U20s and Seniors**

|  |  |  |  |  | 95% CI | |
| --- | --- | --- | --- | --- | --- | --- |
| GPS Variable | Position type | Estimate | *t* | *p* | Lower | Upper |
| M•min^-1^ | Forwards | 1.25 | 0.75 | 0.46 | -2.08 | 4.58 |
|  | Backs | 7.93 | 4.62 | 0.00 | 4.52 | 11.34 |
| HSR m•min^-1^ | Forwards | -2.21 | -3.89 | 0.00 | -3.33 | -1.08 |
|  | Backs | 2.33 | 3.91 | 0.00 | 1.15 | 3.51 |
| HML Distance (m) | Forwards | -166.76 | -2.64 | 0.01 | -292.12 | -41.40 |
|  | Backs | 271.81 | 4.12 | 0.00 | 140.66 | 402.96 |
| HML Efforts | Forwards | -7.33 | -1.29 | 0.20 | -18.60 | 3.95 |
|  | Backs | 22.48 | 3.81 | 0.00 | 10.75 | 34.21 |
| Accelerations 2-3m•s^-^² | Forwards | -5.73 | -2.67 | 0.01 | -9.99 | -1.47 |
|  | Backs | 2.13 | 0.96 | 0.34 | -2.30 | 6.56 |
| Accelerations 3-4m•s^-^² | Forwards | -3.12 | -4.45 | 0.00 | -4.51 | -1.73 |
|  | Backs | -0.19 | -0.26 | 0.80 | -1.62 | 1.24 |
| Accelerations >4m•s^-^² | Forwards | 0.06 | 0.28 | 0.78 | -0.38 | 0.50 |
|  | Backs | 0.40 | 1.78 | 0.08 | -0.05 | 0.85 |
| Decelerations 2-3m•s^-^² | Forwards | -1.41 | -0.72 | 0.47 | -5.31 | 2.49 |
|  | Backs | 3.19 | 1.58 | 0.12 | -0.82 | 7.21 |
| Decelerations 3-4m•s^-^² | Forwards | -2.16 | -2.50 | 0.01 | -3.87 | -0.44 |
|  | Backs | 1.90 | 2.14 | 0.04 | 0.13 | 3.66 |
| Decelerations >4m•s^-^² | Forwards | -1.11 | -2.01 | 0.05 | -2.20 | -0.02 |
|  | Backs | 1.03 | 1.83 | 0.07 | -0.09 | 2.15 |
| Sprint•min^-1^ | Forwards | -0.08 | -3.94 | 0.00 | -0.12 | -0.04 |
|  | Backs | 0.08 | 3.73 | 0.00 | 0.04 | 0.13 |

**Table D. Estimates of fixed effects for all GPS variables displaying difference for position group between U20s and Seniors**

|  |  | Estimate | t | Sig. | 95% Confidence Interval | |
| --- | --- | --- | --- | --- | --- | --- |
| GPS variable | Positional group |  |  |  | Lower Bound | Upper Bound |
| M•min^-1^ | FR | -4.44 | -1.69 | 0.09 | -9.66 | 0.77 |
|  | SR | 3.04 | 1.08 | 0.28 | -2.52 | 8.60 |
|  | BR | 4.51 | 1.92 | 0.06 | -0.17 | 9.19 |
|  | HB | 12.14 | 4.29 | 0.00 | 6.49 | 17.80 |
|  | MF | 6.81 | 2.45 | 0.02 | 1.27 | 12.35 |
|  | B3 | 5.85 | 2.45 | 0.02 | 1.12 | 10.58 |
| HSR m•min^-1^ | FR | -3.38 | -3.79 | 0.00 | -5.15 | -1.61 |
|  | SR | -2.02 | -2.12 | 0.04 | -3.92 | -0.13 |
|  | BR | -1.27 | -1.49 | 0.14 | -2.98 | 0.43 |
|  | HB | 1.27 | 1.18 | 0.24 | -0.88 | 3.43 |
|  | MF | 3.05 | 3.02 | 0.00 | 1.05 | 5.05 |
|  | B3 | 2.45 | 2.96 | 0.00 | 0.81 | 4.09 |
| Accelerations 2-3m•s^-^² | FR | -14.59 | -4.47 | 0.00 | -21.08 | -8.10 |
|  | SR | -2.29 | -0.66 | 0.51 | -9.22 | 4.63 |
|  | BR | -0.74 | -0.25 | 0.81 | -6.77 | 5.29 |
|  | HB | 2.50 | 0.68 | 0.50 | -4.94 | 9.95 |
|  | MF | 4.09 | 1.15 | 0.26 | -3.02 | 11.21 |
|  | B3 | 0.47 | 0.16 | 0.88 | -5.48 | 6.42 |
| Accelerations 3-4m•s^-^² | FR | -4.09 | -3.60 | 0.00 | -6.35 | -1.84 |
|  | SR | -3.31 | -2.73 | 0.01 | -5.72 | -0.91 |
|  | BR | -2.20 | -2.13 | 0.04 | -4.26 | -0.14 |
|  | HB | -0.34 | -0.28 | 0.79 | -2.86 | 2.17 |
|  | MF | -1.31 | -1.07 | 0.29 | -3.74 | 1.13 |
|  | B3 | 0.70 | 0.68 | 0.50 | -1.36 | 2.76 |
| Accelerations >4m•s^-^² | FR | -0.18 | -0.49 | 0.63 | -0.90 | 0.55 |
|  | SR | 0.30 | 0.77 | 0.44 | -0.47 | 1.07 |
|  | BR | 0.09 | 0.28 | 0.78 | -0.56 | 0.74 |
|  | HB | 0.57 | 1.45 | 0.15 | -0.21 | 1.36 |
|  | MF | 0.44 | 1.14 | 0.26 | -0.33 | 1.21 |
|  | B3 | 0.26 | 0.79 | 0.43 | -0.39 | 0.92 |
| Decelerations 2-3m•s^-^² | FR | -12.29 | -4.43 | 0.00 | -17.78 | -6.79 |
|  | SR | -0.29 | -0.10 | 0.92 | -6.15 | 5.58 |
|  | BR | 6.60 | 2.71 | 0.01 | 1.74 | 11.46 |
|  | HB | 6.03 | 2.07 | 0.04 | 0.20 | 11.86 |
|  | MF | 5.97 | 2.07 | 0.04 | 0.21 | 11.73 |
|  | B3 | -0.91 | -0.37 | 0.72 | -5.88 | 4.06 |
| Decelerations 3-4m•s^-^² | FR | -4.87 | -3.61 | 0.00 | -7.55 | -2.20 |
|  | SR | -2.46 | -1.71 | 0.09 | -5.32 | 0.39 |
|  | BR | 0.24 | 0.20 | 0.84 | -2.19 | 2.68 |
|  | HB | 1.26 | 0.85 | 0.40 | -1.70 | 4.22 |
|  | MF | 3.12 | 2.16 | 0.03 | 0.24 | 5.99 |
|  | B3 | 1.40 | 1.14 | 0.26 | -1.04 | 3.83 |
| Decelerations >4m•s^-^² | FR | -2.34 | -2.65 | 0.01 | -4.09 | -0.59 |
|  | SR | -0.73 | -0.78 | 0.44 | -2.60 | 1.14 |
|  | BR | -0.38 | -0.47 | 0.64 | -1.97 | 1.22 |
|  | HB | 0.24 | 0.25 | 0.80 | -1.70 | 2.18 |
|  | MF | 0.67 | 0.71 | 0.48 | -1.21 | 2.56 |
|  | B3 | 1.79 | 2.23 | 0.03 | 0.20 | 3.39 |
| HML Distance (m) | FR | -429.55 | -4.52 | 0.00 | -618.18 | -240.93 |
|  | SR | -104.48 | -1.03 | 0.31 | -305.81 | 96.84 |
|  | BR | 21.03 | 0.23 | 0.82 | -157.91 | 199.97 |
|  | HB | 280.12 | 2.50 | 0.02 | 56.47 | 503.78 |
|  | MF | 332.82 | 3.15 | 0.00 | 122.37 | 543.28 |
|  | B3 | 225.54 | 2.57 | 0.01 | 51.45 | 399.62 |
| Sprint•min^-1^ | FR | -0.13 | -4.08 | 0.00 | -0.20 | -0.07 |
|  | SR | -0.07 | -2.10 | 0.04 | -0.14 | 0.00 |
|  | BR | -0.04 | -1.39 | 0.17 | -0.11 | 0.02 |
|  | HB | 0.03 | 0.64 | 0.53 | -0.05 | 0.10 |
|  | MF | 0.11 | 2.97 | 0.00 | 0.04 | 0.18 |
|  | B3 | 0.09 | 3.17 | 0.00 | 0.04 | 0.15 |
| HML Efforts | FR | -35.98 | -4.49 | 0.00 | -51.90 | -20.07 |
|  | SR | -1.06 | -0.13 | 0.90 | -18.04 | 15.91 |
|  | BR | 13.31 | 1.81 | 0.08 | -1.38 | 28.01 |
|  | HB | 37.78 | 4.20 | 0.00 | 19.72 | 55.84 |
|  | MF | 24.40 | 2.81 | 0.01 | 7.07 | 41.74 |
|  | B3 | 11.93 | 1.63 | 0.11 | -2.63 | 26.49 |
